# Supplementary material for: Development of a Nomogram Model for Treatment of Elderly Patients with Locoregionally Advanced Nasopharyngeal Carcinoma
Source: J Pers Med. 2021 Oct 22;11(11):1065. doi: 10.3390/jpm11111065 (PMC8618502; doi:10.3390/jpm11111065)

INDEX OF SUPPLEMENTARY INFORMATION

SUPPLEMENTARY MATERIALS AND METHODS.……………..….Page 1

SUPPLEMENTARY RESULTS………..…….………………………......Page 3

SUPPLEMENTARY TABLES………..…….……………………....….Page 4

SUPPLEMENTARY FIGURES………..……..…………………………..Page 7

SUPPLEMENTARY MATERIALS AND METHODS

Examinations

All patients underwent baseline examinations before initiating treatment, including medical history, physical examination, biochemistry and hematology profiles, quantitative analysis of cf EBV DNA, fiberoptic nasopharyngoscopy, magnetic resonance imaging (MRI) with contrast of the suprasellar cistern to the collarbone, chest radiography, abdominal sonography, and bone scintigraphy.

The quantitative cf EBV DNA assay was conducted in the Department of Molecular Diagnosis at SYSUCC. Our laboratory is experienced in measuring cf EBV DNA load, and was certified by Stanford University as part of the EBV DNA-guided international NRG-HN001 trial for NPC [ClinicalTrials.gov identifier: NCT02135042]. Samples of peripheral venous blood (5 ml) were obtained from all patients before treatment initiation. For isolation of plasma and peripheral blood cells, the bloods were placed in ethylene diaminete traacetic acidecontaining tubes and centrifuged at 1600 g for 15 minutes. Plasma DNA were extracted using the QIAamp Blood Kit (Qiagen, Hilden, Germany). EBV DNA concentrations were detected toward the BamHI-W region of the EBV genome using a real-time PCR assay, as previously described [1]. The sequences of the forward and reverse primers used in the assay were 5`-GCCAG AGGTA AGTGG ACTTT-3` and 5`-TACCA CCTCC TCTTC TTGCT-3`. The dual fluorescently-labelled oligomer of 5`-(FAM) CACAC CCAGG CACAC ACTAC ACAT (TAMRA)-3` served as the probe.

Treatment

The specific chemotherapy regimen was based on the experience of the physician. Of the 583 patients who met the inclusion criteria, all received external beam RT by conventional fractionation and 58.8% (343/583) of patients received radiotherapy combined with platinum-based chemotherapy (CRT). Of the total patients, 97.6% (569/583) of patients received IMRT,

and only 13 (2.2%) and 1 (0.2%) patients received conventional 2D and 3D conformal RT. Of the 343 patients who received CRT, 44.9% (154/343) of patients received concurrent chemoradiotherapy (CCRT), 34.1% (117/343) of patients received induction chemotherapy (IC) followed by CCRT, and 21.0% (72/343) of patients received IC followed by RT. For those who received IC (*n* =188), more than half received doublet IC regimes (*n* =130), including docetaxel-cisplatin (TP) (*n* =73), cisplatin-fluorouracil (PF) (*n* = 48), gemcitabine-cisplatin (GP) (*n* =6) and pemetrexed-cisplatin (AP) (*n* =3). Another 55 patients received triplet docetaxel-cisplatin-fluorouracil (TPF) IC and 3 received oral 5-fluorouracil single-agent IC. For the number of IC cycles, the majority of patients received 2 to 4 cycles of IC (*n* =160), while only 28 patients received 1 cycle of IC. For those who received CCRT (*n* =280), cisplatin was the most common drug used in combination with radiotherapy (*n* =174); nedaplatin (*n* =32) was the other concurrent systemic agent. Other concurrent chemotherapy drugs included loplatin, oxaliplatin, carboplatin, 5-fluorouracil, docetaxel, cisplatin–fluorouracil (PF) and docetaxel–cisplatin (TP).

Reference

[1] Shao JY, Li YH, Gao HY, et al. Comparison of plasma Epstein-Barr virus (EBV) DNA levels and serum EBV immunoglobulin A/virus capsid antigen antibody titers in patients with nasopharyngeal carcinoma. Cancer 2004, 100(6):1162-1170.

SUPPLEMENTARY RESULTS

The cause of death

In this study, among 196 patients who died during the follow-up period, 65.3% of these patients died of NPC (recurrent, metastatic or residual disease), 11.2% died of therapeutic toxicity; and other causes including accidents (4.1%), cardiovascular diseases (3.6%), secondary cancers (3.1%), gastrointestinal diseases (1.0%), neurological diseases (0.5%) and respiratory diseases (0.5%). The remainder (6.6%) died of unknown causes with the absence of documented relapse.

SUPPLEMENTARY TABLES

Table S1. Point assignment of the nomogram for disease-free survival.

Variables and probability of mortality	Score
T classification [1]	
T1	0
T2	28
T3	54
T4	100
N classification [1]	
N0	0
N1	6
N2	60
N3	60
ALB, g/L[2]	
≤ 40	46
> 40	0
EBV DNA, copies/mL[2]	
< 4000	0
≥ 4000	94
3-year DFS (%)	
50	235
60	198
70	152
75	127
80	95
85	55
5-year DFS (%)	
50	192
60	152
70	108
75	80
80	51

Abbreviations: DFS, disease-free survival; EBV, Epstein-Barr virus; ALB, albumin;

[1] According to the 8th edition of the AJCC/UICC staging system;

[2] All variables were measured before treatment.

Table S2. Baseline characteristics of nomogram-defined high-risk and low-risk groups.

Characteristics	Low-risk group (n = 283) No. (%)	High-risk group (n = 300) No. (%)	P-value [5]
Age, years			0.627
≤ 68	166 (58.7%)	170 (56.7)	
> 68	117 (41.3%)	130 (43.3)	
Gender			0.878
Male	224 (79.2%)	239 (79.7)	
Female	59 (20.8%)	61 (20.3)	
Histological type (WHO) [1]			0.900
Type I-II	9 (3.2)	9 (3.0)	
Type III	274 (96.8)	291 (97.0)	
Smoking			0.815
Yes	114 (40.3)	182 (60.7)	
No	169 (59.7)	118 (39.3)	
Drinking			0.095
Yes	52 (18.4)	260 (86.7)	
No	231 (81.6)	40 (13.3)	
Family history of NPC			0.508
Yes	59 (20.8)	244 (81.3)	
No	224 (79.2)	56 (18.7)	
EBV DNA, copies/mL [2]			< 0.001
< 4000	283 (100%)	37 (12.3)	
≥ 4000	0 (0)	263 (87.7)	
LDH, IU/L [2]			0.001
≤ 250	273 (96.5)	267 (89.0)	
> 250	10 (3.5)	33 (11.0)	
HGB, g/L [2]			0.048
≤ 120	18 (6.4)	33 (11.0)	
> 120	265 (93.6)	267 (89.0)	
CRP, mg/L [2]			0.001
≤ 3	190 (67.1)	159 (53.0)	
> 3	93 (32.9)	141 (47.0)	
ALB, g/L [2]			< 0.001
≤ 40	26 (9.2)	91 (30.3)	
> 40	57 (90.8)	209 (69.7)	
T classification [3]			< 0.001
T1	7 (2.5)	12 (4.0)	
T2	4 (1.4)	14 (4.7)	
T3	237 (83.7)	148 (49.3)	
T4	35 (12.4)	126 (42.0)	
N classification [3]			< 0.001
N0	70 (24.7)	22 (7.3)	

N1	155 (54.8)	110 (36.7)	
N2	46 (16.3)	92 (30.7)	
N3	12 (4.2)	76 (25.3)	
Overall stage [3]			< 0.001
III	235 (83.0)	123 (41.0)	
IVa	48 (17.0)	177 (59.0)	
CCI [4]			0.250
= 2	114 (40.3)	138 (46.0)	
> 2	169 (59.7)	162 (54.0)	
Treatment modality			< 0.001
RT	129 (45.6)	111 (37.0)	
CRT	154 (54.4)	189 (63.0)	

Abbreviations: No., number; WHO, World Health Organization; CCI, Charlson Comorbidity Index; EBV, Epstein-Barr virus; LDH, serum lactate dehydrogenase level; HGB, hemoglobin; CRP, C-reactive protein; ALB, albumin; RT, radiotherapy; CRT, radiotherapy combined with chemotherapy;

[1] WHO Type I, keratinizing, WHO Type II, non-keratinizing (differentiated), WHO Type III, non-keratinizing (undifferentiated);

[2] All variables were measured before treatment;

[3] According to the 8th edition of the AJCC/UICC staging system;

[4] For every decade the patient was over 40 years old 1 point was added to risk and the “age points” added to the Charlson Comorbidity Index. Therefore, no patient in our study had a CCI score below 2;

[5] *P*-values were calculated by Pearson $\chi 2$ test or Fisher’s exact test.

SUPPLEMENTARY FIGURES

Figure S1. Receiver operating characteristic (ROC) curves of the nomogram, pretreatment EBV DNA load, the 8th edition of the AJCC/UICC TNM staging system, T classification, N classification and ALB for predicting DFS (using MedCalc software). EBV, Epstein-Barr virus; ALB, albumin; DFS, disease-free survival.

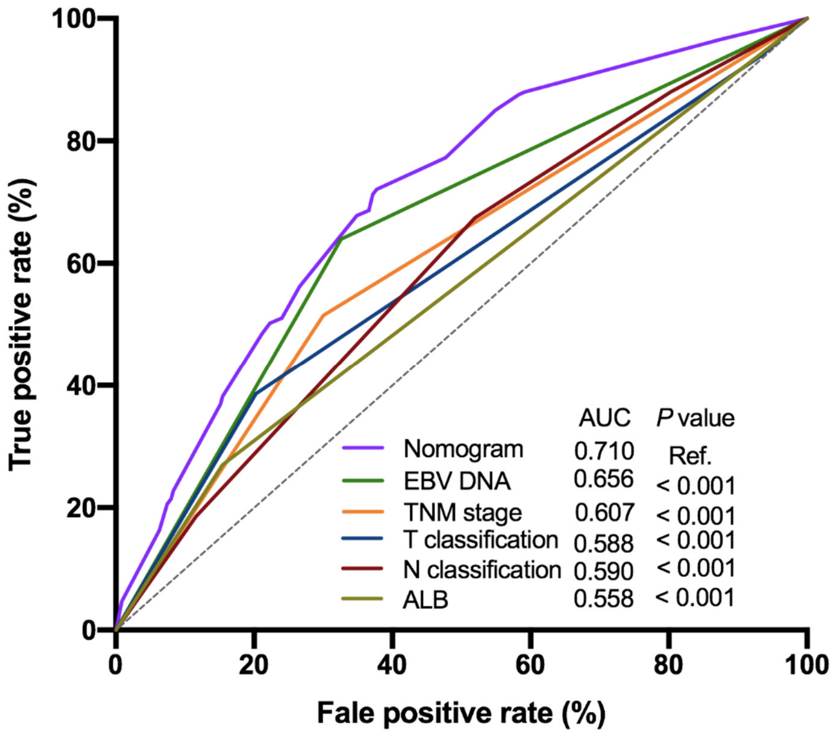

Figure S2. X-tile analysis to determine the optimal cutoff value of the nomogram for survival prediction in elderly patients with LA-NPC. Red coloration of cut-points indicates an inverse correlation with survival, whereas, green coloration represents direct associations. Diffuse red indicates a continuous indirect association between increasing tumor size and good prognosis. The cutoff value determined by X-tile analysis in the left panel (a) is shown on a histogram of the entire cohort (b), and a Kaplan-Meier plot (c; low-risk group =blue, high-risk group =gray). LA-NPC, locoregionally advanced nasopharyngeal carcinoma.

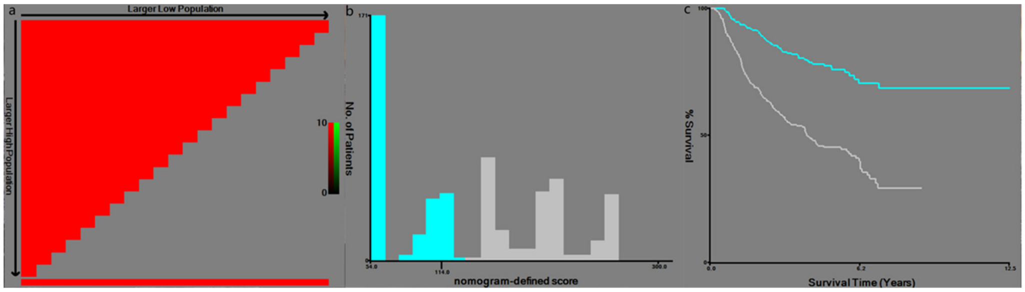

Supplement: Supplementary file 1 [file jpm-11-01065-s001.zip › jpm-1416535 -supplementary.pdf]
